# Psychiatric Co-Morbidities in Pregnant Women with Opioid Use Disorders: Prevalence, Impact, and Implications for Treatment

**DOI:** 10.1007/s40429-017-0132-4

**Published:** 2017-03-09

**Authors:** Camila L. Arnaudo, Barbara Andraka-Christou, Kacy Allgood

**Affiliations:** 1 Department of Psychiatry, Indiana University School of Medicine, 355 West 16th Street, Suite 2800, Indianapolis, IN 46202 USA; 2 Department of Health Policy and Management, Indiana University, Richard M. Fairbanks School of Public Health, Indianapolis, IN USA; 3 Ruth Lilly Medical Library, Indiana University, Indianapolis, IN USA

**Keywords:** Pregnant, Dual diagnosis, Comorbidity, Substance use disorder, Addiction, Opioid, Psychiatric disorder

## Abstract

**Purpose of Review:**

This review seeks to investigate three questions: What is the prevalence of comorbid psychiatric diagnoses among pregnant women with opioid use disorder (OUD)? How do comorbid psychiatric illnesses impact pregnant women with OUD? And how do comorbid psychiatric illnesses affect the ability of pregnant women with OUD to adhere to and complete OUD treatment?

**Recent Findings:**

Based on this literature review, 25–33% of pregnant women with OUD have a psychiatric comorbidity, with depression and anxiety being especially common. However, of the 17 studies reviewed only 5 have prevalence rates of dual diagnosis in pregnant women with OUD as their primary outcome measures, their N’s were typically small, methods for determining psychiatric diagnosis were variable, and many of the studies were undertaken with women presenting for treatment which carries with its implicit selection bias. Of the women enrolled in treatment programs for SUD, those with psychiatric comorbidity were more likely to have impaired psychological and family/social functioning than those without psychiatric comorbidity. Greater severity of comorbid psychiatric illness appears to predict poorer adherence to treatment, but more research is needed to clarify this relationship with the psychiatric illness is less severe.

**Summary:**

While cooccurrence of psychiatric disorders in pregnant women with opioid use disorder appears to be common, large population-based studies with validated diagnostic tools and longitudinal assessments are needed to obtain definitive rates and characteristics of cooccurring illnesses. Integrated prenatal, addiction, and psychiatric treatment in a setting that provides social support to pregnant patients with OUD is most effective in maintaining women in treatment. More research is still needed to identify optimal treatment settings, therapy modalities, and medication management for dually diagnosed pregnant women with OUD.

## Introduction

Misuse of opioids, both prescription pain medication and heroin, is a public health crisis in the USA that is linked to skyrocketing morbidity and mortality rates, health care costs, criminal justice expenditures, and productivity loss. The rate of increase in opioid misuse has been particularly acute in women of reproductive age, producing a surge in opioid use disorder (OUD) among pregnant women [1]. A study of over 57 million births in the USA between 1998 and 2011 found a 127% increase in the rate of women diagnosed with opioid abuse or dependence at the time of delivery [2]. As compared to men with OUD, women with OUD are more likely to be younger, victims of emotional, physical, or sexual abuse, experiencing other mental health concerns (including depression and anxiety), and prescribed psychiatric medications [3].

Medical providers and other treatment experts regard pregnancy as an especially important time for medical intervention in substance use disorder (SUD), often motivating women to make lifestyle changes and reduce substance use. For example, the prevalence of cigarette smoking declines from 23% among women before pregnancy to 15% during pregnancy, alcohol use declines from 55 to 10%, and illicit substance use declines from 10 to 5% [4]. Women who continue to use substances during their pregnancies face several barriers to achieving sobriety. External barriers to treatment include inadequate finances, lower educational achievement, lack of childcare, limited engagement with the labor market, and long wait for appointments [5]. Studies have found that pregnant women have relatively high dropout rates even in studies of programs providing intensive psychosocial support [6]. This suggests that there may be other barriers that pregnant women with OUD are facing, and studies have found internal barriers of shame, fear of judgment by health care providers, fear of referral to social services, and losing custody of children [5].

Rates of mood and anxiety disorder have been found to increase during pregnancy and thus may also be higher in pregnant women with OUD compared to nonpregnant women with OUD, and this may be an additional factor influencing pregnant women’s ability to engage in treatment [7]. Patients with non-SUD psychiatric illness have impairments in neurobiological pathways like those in patients with SUD, which predispose patients with non-SUD’s to developing a SUD and in some ways, complicates these patients’ illness course [8–10].

Researchers currently have only a limited understanding of the prevalence and variety of psychiatric comorbidity in pregnant women with OUD and the effect of dual diagnosis on illness severity and treatment outcomes. Even comprehensive care models for pregnant women with OUD, such as programs with antenatal care, may not incorporate psychiatric assessment as part of their intake process [11, 12]. Extensive research has gone into investigating the safety for mother and fetus of using Medication Assisted Treatment (MAT), but little guidance can be found on what psychiatric medication to use to treat the cooccurring illnesses that these women face while pregnant [13].

To broaden this knowledge, the authors performed a literature review of current published studies that address OUD in pregnant women and selected studies of comorbidity prevalence rates and treatment impact for closer evaluation. Three research questions were asked. First, how prevalent are comorbid psychiatric diagnoses among pregnant women with OUD? Second, how do comorbid psychiatric illnesses impact the illness severity in pregnant women with OUD? Finally, how do comorbid psychiatric illnesses impact treatment adherence in pregnant women with OUD? The answers to these research questions could be used to improve treatment programs and thus clinical outcomes, function, and health among an understudied, vulnerable population. Based on these results, the authors proffer recommendations for treatment programs and suggestions for future research.

## Methods

In August 2016, the authors searched Ovid MEDLINE(R), Ovid MEDLINE(R) Daily Update, and Ovid MEDLINE (R) In-Process & Other Non–Indexed Citations for subject (MeSH) headings for pregnancy, opioid treatment, opioid-related disorders, demography, and geographical location. The search returned a total of 914 articles (see Appendix for complete strategy), starting in 1946. Non-English language publications, comments, and animal studies were excluded, resulting in 719 articles. Importantly, of these 719 articles, 230 focused on neonatal abstinence syndrome, birth outcomes, or childhood outcomes rather than on the mother’s health. Articles that did not discuss maternal health were manually excluded leaving a total of 489 articles. From this group of 489 articles, the following were manually excluded: nonoriginal research studies (e.g., guidance documents without original results), studies that were literature reviews and case reports/series with sample sizes of less than 30, and studies that did not examine the intersection of OUD and comorbid psychiatric disorders in pregnant women, leaving a total of 31 articles. Three further studies were included, which had subjects with OUD but reported aggregate results for all patients with SUD (including patients with and without opioid use disorder). Included, for example, is a study by Haller et al. in which only 9% of subjects with SUD had OUD, because few other studies discussed personality disorders and psychotic illnesses among pregnant women with SUD [14]. Results from Moylan et al.’s study, in which 56% of subjects were enrolled in methadone maintenance programs, and results from Eggleston et al.’s study, in which 89% of subjects had OUD diagnosis, were also included in the review [15, 16••].

## Results

### Prevalence

The authors found 21 studies discussing the prevalence of comorbid psychiatric disorders in pregnant women with OUD. They excluded four of these studies for the following reasons. One study (Gordon et al. 2012) avoided patients with mental health conditions whose medications might interact with maintenance therapy or affect pregnancy outcomes, potentially biasing prevalence rates, two studies (Lacroix et al. 2011; Lund et al. 2013) examined prevalence of prescriptions for comorbid psychiatric conditions rather than diagnoses, and one study (Perry et al. 2015) had a small sample size (*n* = 15) [17–20]. After excluding these studies, our final analysis contained 17 studies discussing the prevalence of comorbid psychiatric disorders in pregnant women with OUD.

Methodology, sampling strategy, and study design varied greatly among these studies, making comparisons difficult. Settings also varied, both in terms of geography (England, Australia, and the USA) and medical practice environment (hospitals, outpatient clinics, or both). Prevalence of comorbid psychiatric conditions in pregnant women with OUD was a primary study outcome in only five studies (Ordean et al. 2013; Steer and Schut 1980; Holbrook and Kaltenbach 2012; Burns et al. 1985; Benningfield et al. 2010). The combined total sample size for these five studies was 531 participants. Prevalence rates from other studies may be subject to sampling bias due different primary study outcomes. Of the five studies that included comorbid psychiatric conditions as a primary outcome, three reported validated screening measures (Steer and Schut 1980; Burns et al. 1985; Benningfield et al. 2010), but only one study (Benningfield et al. 2010) occurred in the last 10 years.

Psychiatric diagnoses were not always obtained using validated screening tools. Only 10 of the 17 studies discussing prevalence clearly used a validated screening tool during patient interviews (see Table 1). Prevalence rates from 2 of these 10 studies (Benningfield et al. 2010 and Benningfield et al. 2012) should be interpreted with caution: They conducted secondary analysis of a larger study (the Maternal Opioid Treatment: Human Experimental Research (MOTHER study). The MOTHER study had used the Mini International Neuropsychiatric Interview (MINI) to putatively diagnose psychiatric disorders. Additionally, more patients were reported to use mental health medication during the MOTHER study than were diagnosed with mental health conditions at treatment entry, suggesting that prevalence rates are underestimates [17, 18]. Of the remaining studies, three examined only medical records data (Ordean et al. 2013; Patel et al. 2013), two studies used aggregated institutional data reported to the US government (Martin et al. 2015; Terplan et al. 2010; Oei et al. 2009), one study conducted patient interviews without the use of a validated screening tool (Greig et al. 2012), and one study did not clarify how psychiatric diagnoses were obtained (Tuten et al. 2012) [23–25, 26••, 27–29]. Prevalence rates based solely on medical records or institutional reports should be interpreted with caution, since it is impossible to ascertain whether the healthcare provider used a validated screening tool.

**Table 1:** Prevalence of Comorbid Psychiatric Disorders

Authors (publication year) (nation where study took place)	Comorbidity prevalence	Mood disorder prevalence	Anxiety disorder prevalence	Depression prevalence	Anxiety prevalence	PTSD prevalence	Strengths	Limitations
Martin CE, Longinaker N, Terplan M (2015) (USA)	14% in 1992; 31% in 2012						Very large N	Institutionally reported data to US government (unclear screening method)
Ordean A, Kahan M, Graves L, Abrahams R, Boyajian T (2013) (Canada)				45%	20%		3 study sites	Retrospective chart review (unclear screening method)
Patel P, Abdel-Latif ME, Hazelton B, Wodak A, Chen J, Emsley F, Feller JM, Lui K, Oei JL, (2013) (Australia)	45%							Retrospective chart review (unclear screening method)
Steer RA, Schut J, (1980) (USA)		Comparable between pregnant and nonpregnant opioid using women					Cohort study; used validated screening tool	One study site
Tuten M, Svikis DS, Keyser-Marcus L, O’Grady KE, Jones HE (2012) (USA)		58% in last 30 days	42% in last 30 days	46% in last 30 days; 55% lifetime adjusted	31% last 30 days; 38% lifetime adjusted			One study site; unclear screening method
Holbrook A, Kaltenbach K (2012) (US)				30% (at treatment entry); 44% (postpartum)			Longitudinal study (used validated screening tool at time 2)	Unclear screening method at time 1; one study site
Burns K, Melamed J, Burns W, Chasnoff I, Hatcher R (1985) (USA)				50% (60% among older women, 25% among young women, and 8% among teenagers)			Used validated screening tool	Small N (*n* = 54)
Oei JL, Abdel-Latif ME Craig F, Kee A, Austin MP, Lui K (2009) (Australia)	45%			Most common dual diagnosis				Retrospective chart review (unclear screening method)
Greig E, Ash A, Douiri A (2012) (England)	25%			9% at treatment entry; 4.5% postpartum				No use of validated screening tool; one study site; small N (*n* = 44)
Terplan M, Smith EJ, Glavin SH (2010) (USA)	21%							Institutionally reported data to US government (unclear screening method)
Chisolm MS, Tuten M, Brigham EC, Strain EC, Jones HE (2009) (USA)	53%		36%	36%		3%	Used validated screening tool	One study site
Benningfield MM Arria AM, Kaltenbach K, Heil SH, Stine SM, Coyle MG, Fischer G, Jones HE, Martin PR (2010) (USA)	65%	49%	40%	32%	40%	16%	Large N; multisite study; used validate screening tool (MINI)	Secondary analysis of MOTHER study; excluded patients with psychosis
Moylan PL, Jones HE, Haug NA, Kissin WB, Svikis DS (2001) (USA)	32% had cooccurring Axis I disorder; 22% had cooccurring axis 2 disorder					19% (having PTSD doubled chance of axis I or II disorder)	Used validated screening tool	One study site
Eggleston AM, Calhoun PS, Svikis DS, Tuten M, Chisolm MS, Jones HE (2009) (USA)	41% had cooccurring Axis 1 disorder (excluding PTSD)			38%		26% (having PTSD increased likelihood of panic disorder or social anxiety disorder)	Used validated screening tool	One study site
Benningfield MM, Dietrich MS, Jones HE, Kaltenbach K, Heil SH, Stine SM, Coyle MG, Arria AM, O’Grady KE, Fischer G, Martin PR (2012) (USA)	62% (32% had both MDD and anxiety)					19%	Large N; multisite study; used validated screening tool (MINI)	Secondary analysis of MOTHER study; excluded patients with psychosis
Fitzsimons HE, Tuten M, Vaidya V, Jones HE (2007) (US)	72%	27%	36%				Used validated screening tool	One study site
Tuten M, Heil SH, O’Grady KE, Fitzsimons H, Chisolm MS, Jones HE (2009) (USA)		56% (44% with mood disorder also had anxiety disorder)					Used validated screening tool (in original study)	Secondary analysis of Fitzsimons et al. 2007 study

Reported rates are at treatment entry, unless otherwise stated. If study used medical records, the authors do not know when screening occurred

Other factors also bespeak caution. Many studies assessed psychiatric conditions before OUD treatment began (see Table 1); however, previous research has demonstrated that certain psychiatric symptoms may diminish after OUD treatment commences. In contrast, some studies obtained prevalence rates after OUD treatment had begun. Ideally, studies should assess prevalence longitudinally. One study examined prevalence longitudinally (Greig et al. 2012), but used medical records to assess prevalence in time 1 and used a validated screening tool in time 2, limiting the longitudinal results’ reliability [19].

Studies reported high rates of comorbid psychiatric disorders in pregnant women with OUD, with rates ranging from 72 to 21%. Fitzimons et al. found a 72% prevalence rate, Benningfield et al. and Eggleston each found prevalence rates of over 60%, Oei et al. and Patel et al. each found a 45% prevalence rate, Martin et al. found a 31% prevalence rate, Greig et al. found a 25% prevalence rate, and Terplan et al. found a 21% prevalence rate [16••, 22••, 23–25, 26••, 27, 28]. Moylan et al. found a prevalence rate of 32% for Axis I disorders and 23% for Axis II disorders. Furthermore, the rate of pregnant women with OUD with at least one comorbid psychiatric disorder more than doubled since 1992 [15, 20].

Only a few studies compared prevalence rates between groups. Using a cross-sectional study, Steer and Schut compared the rate of mood disorders between pregnant women with OUD and nonpregnant women with OUD, but did not find a significant difference in prevalence rates between the two groups. One study (Oei et al. 2009) found that pregnant women with OUD and poly-substance use were more likely to have comorbid psychiatric disorders than pregnant women with OUD but without poly-substance use, and rates of nicotine use were not statistically significantly different between the two groups (83.6 vs. 81.3%, *p* = 0.42) [21]. Chisolm et al. and Eggleston et al. found that pregnant women with SUD who smoked cigarettes were more likely to have comorbid psychiatric disorders than nonsmoking pregnant women with SUD (although almost 90% of the study sample smoked cigarettes) [22••]. Finally, in a cross-sectional study using medical records, Patel et al. found no statistically significant difference in rates of comorbid psychiatric conditions between pregnant women with OUD undergoing buprenorphine treatment, pregnant women with OUD not undergoing buprenorphine treatment, and pregnant women with a nonopioid substance use disorder [23].

Mood disorders are the most commonly reported comorbid psychiatric disorders in pregnant women with OUD. Tuten et al. reported a comorbid mood disorder rate of 58%, Tuten reported a 56% comorbid mood disorder rate in methadone-stabilized patients at treatment entry, and Fitzimons et al. reported a 27% comorbid mood disorder rate in methadone-stabilized patients at treatment entry [24, 25, 26••]. In the studies reviewed, depression was the most commonly reported mood disorder. Burns et al., Ordean et al., and Tuten et al. each found a comorbid depression prevalence rate of around 50%, and Eggleston et al. found a rate of 38% [16••, 24, 27]. Interestingly, Burns et al. found that older pregnant women with SUD exhibited significantly higher rates of depression than did younger pregnant women with SUD. Greig et al. found that antenatal depression predicted postnatal depression, although the study’s methodology may have underestimated the depression rate at treatment entry [19].

Anxiety disorders were the second most commonly reported comorbid psychiatric disorders in pregnant women with OUD. Tuten et al. found an anxiety prevalence rate of 42% in this population, Benningfield et al. found a prevalence rate of 40%, and Fitzimmons et al. found a prevalence rate of 36% [18, 24, 26••]. Multiple studies reported prevalence rates of posttraumatic stress disorder (PTSD) in pregnant women with OUD, Eggleston et al. found a 26% PTSD prevalence rate, Moylan et al. found a 19% prevalence rate, Benningfield et al. found a 16% prevalence rate, and Chisolm et al. found a 3% prevalence rate. Minimal discussion of comorbid psychosis, bipolar disorder, obsessive compulsive disorders, or eating disorders existed in the literature [15, 16••, 22••]. Eggleston et al. reported a 6% prevalence of bipolar disorder and a 2% prevalence rate of eating disorder among pregnant women with OUD at treatment entry. Notably, multiple studies excluded participants with symptoms of psychosis [16••].

Pregnant women with OUD frequently have more than one comorbid psychiatric disorder. Tuten et al. reporting that 44% of pregnant women with OUD had a mood disorder and an anxiety disorder [25]. PTSD also frequently occurred with other psychiatric disorders. In a cross-sectional study, Moylan et al. found that pregnant women with both OUD and PTSD were twice as likely to have an additional comorbid Axis I lifetime mood disorder (50 vs. 27%, *P* < .05) and twice as likely to have an additional comorbid Axis II lifetime personality disorder as compared to pregnant women with OUD but without PTSD [15]. In a cross-sectional study, Eggleston et al. found that pregnant women with OUD and PTSD exhibited higher rates of current panic disorder and social anxiety disorder than did pregnant women with OUD but without PTSD [16••].

### Impact of Dual Diagnosis on Pregnant Women Diagnosed with Opioid Use Disorder

Of over 700 articles identified by our literature search, only seven (Table 2) met our search criteria as research studies exploring the impact of dual diagnosis on pregnant women with OUD [14–18, 22••, 26••]. The seven articles primarily discussed the impact of the following psychiatric diagnoses on pregnant women with OUD: mood disorders (mostly depressive illnesses), anxiety disorders, and PTSD. To establish diagnoses, four of the studies conducted Structured Clinical Interview for DSM-IV (SCID) interviews, which is the gold standard for determining formal psychiatric diagnosis in research studies [15, 16••, 22••, 26••]. Two studies used the MINI instead of the longer SCID, the limitations of which are discussed above. One study by Haller et al. used the Minnesota Multiphasic Personality Inventory (MMPI), Structured Interview for DSM-III-R Personality Disorders (SIPD-R), and Millon Clinical Multiaxial Inventory (MCMI-II) self-report questionnaires to aid in determining diagnoses and illness severity; the researchers stated that they conducted standardized interviews for Axis I disorders but did not detail how these were conducted [14].

**Table 2:** Impact of cooccurring psychiatric disorders

Author (year of publication)	Title	Method	Question	Results	Limitations
Fitzsimons HE, Tuten M, Vaidya V, Jones HE (2007)	Mood disorders affect drug treatment success of drug-dependent pregnant women	Cohort; (*N* = 106)	What is the impact of cooccurring Axis I disorders on drug treatment outcomes of drug-dependent pregnant women?	Mood Disorder (MD) and Anxiety Disorder (AD) groups had higher incidence of psychosocial impairment and suicidal ideation; AD spent more time in treatment than MD or No Diagnosis (ND) group; MD group had more positive urine screens than AD and ND groups; confirms previous findings that MD poorly affects treatment outcomes; new finding that Ad did not negatively affect tx; ASI scores similar in AD, MD and ND groups over 5 domains, MD and AD groups had higher scores on psychiatric and family/social hx composite scores.	Recall bias; portion of participants left prior to data collection, may limit generalizability; no tracking of psych symptoms, only initial assessment; symptom overlap with 44% of MD having anxiety and 35% of AD group having depression;
Benningfield MM, Dietrich MS, Jones HE, Kaltenbach K, Heil SH, Stine SM, Coyle MG, Arria AM, O’Grady KE, Fischer G, Martin PR (2012)	Opioid dependence during pregnancy: relationships of anxiety and depression symptoms to treatment outcomes.	Secondary Analysis of an RCT (*N* = 175)	What is the relationship of anxiety and depression with treatment outcome in opioid-dependent pregnant women? What are their psychotropic medications?	In terms of treatment attrition AD(47%) > AD + MD (25%) = ND (18.2%) > MD (10.5%) No difference were observed with ongoing illicit drug use or pre-term delivery; ASI psychological composite score was higher for AD, MD, and AD + MD, than ND group; no significant association found between ASI score and discontinuation of tx; a trend was found toward more urines with illicit substances and the MD + AD group, but it was not statistically significant; no statistically significant association between psychiatric symptoms and pre-term delivery	Only brief screening tool for psych symptoms; so no confirmative psych diagnosis; only initial screening; threshold for anxiety and depression symptoms lower that what is needed for formal dx; no longitudinal measure; psychosocial treatments were not standardized; secondary analysis makes statistical power limited, results should be considered exploratory; psychiatric tx not necessary provided by study staff
Haller DL, Miles DR, Dawson KS (2002)	Psychopathology influences treatment retention among drug-dependent women.	Cohort (*N* = 78)	What is the relationship between psychiatric morbidity (including personality disorders) and treatment drug out in a group of pregnant drug dependent women?	Study controlled for structural barriers that impede program participation; Group of patients with most severe psychopathology had highest dropout rate, even though they did not have most severe substance use disorder diagnosis. Group with significant substance dependence and predominant borderline personality disorder had the highest retention rate compared with group with little psychopathology and group with more severe psychopathology.	Use self-report tools to evaluate symptoms and diagnoses; did not use SCID “gold standard” to get DSM diagnoses; Only 9% of patients abusing opiates, most 87% abusing cocaine; only 9% opioids
Eggleston AM, Calhoun PS, Svikis DS, Tuten M, Chisolm MS, Jones HE (2009)	Suicidality, aggression, and other treatment considerations among pregnant, substance-dependent women with posttraumatic stress disorder.	Cross-sectional (*N* = 105)	What is the relationship between PTSD and SUD and does it confer greater risk and severity than what is seen with SUD and other Axis I disorders?	Three study groups evaluated (SUD-PTSD, SUD-PSY and SUD-only) showed equivalent substance use severity, however the SUD-PTSD group had higher odds of reporting suicidality, aggression, and psychosocial impairment (more problematic relationships, more illegal behavior, more homelessness) than both the SUD-PSY and SUD-only groups. SUD-PTSD groups had high rate of other Axis I diagnoses, similar to that in the SUD-PSY group. Rates and numbers of substances used did not differ	Majority of patients had opioid use disorder, 11% had cocaine use disorder and not opioid; small sample size; psychotic illness was excluded; homogeneous sample in terms of substance use severity
Moylan PL, Jones HE, Haug NA, Kissin WB, Svikis DS (2001)	Clinical and psychosocial characteristics of substance-dependent pregnant women with and without PTSD.	Cross-sectional (*N* = 123)	How does the psychiatric and psychosocial functioning of opiate or cocaine dependent pregnant women with and without a diagnosis of PTSD differ?	Women with a diagnosis of PTSD had higher rates of Axis I and Axis II diagnosis than opiate or cocaine dependent women without PTSD. Women with PTSD had higher levels of emotional, physical and sexual abuse, higher rates of suicide attempts, higher ASI impairment on family/social and psychiatric domains. Severity of substance use disorder and group attendance did not differ in women with and without PTSD.	Low rate of PTSD in sample population (19%), which is low compared to other samples with addictions, authors suggest due to assessment being made early in treatment when patients less likely to disclose trauma; only 1 measure used to assess PTSD; Not all women opioid addicted; some cocaine only; 56% were on methadone maintenance
Benningfield MM Arria AM, Kaltenbach K, Heil SH, Stine SM, Coyle MG, Fischer G, Jones HE, Martin PR (2010)	Cooccurring psychiatric symptoms are associated with increased psychological, social, and medical impairment in opioid dependent pregnant women.	Secondary analysis of an RCT (*N* = 174)	What is the relationship of psychiatric symptoms to severity of drug use and drug related problems among pregnant women with opioid use disorder?	Majority of sample (50.3%) screened positive for cooccurring psychiatric illnesses, these patients had increased psychiatric and social impairment as measured by ASI; the impaired domains on ASI varied by cooccurring diagnosis; the drug domain was more impaired in subjects diagnosed with MDD and Dysthymia but not other illnesses; the sample showed a low prevalence for PTSD of only 16% and high prevalence of hypomania of 39%	MINI screener used to establish symptoms and diagnosis with no SCID, so the meaning of rates of illness are not well established; only symptoms at intake were measured and no longitudinal assessment of how these progressed during pregnancy; Axis II disorders not evaluated
Chisolm MS, Tuten M, Brigham EC, Strain EC, Jones HE (2009)	Relationship between cigarette use and mood/anxiety disorders among pregnant methadone-maintained patients.	Cross-Sectional (*N* = 112)	What is the association between cigarette smoking during pregnancy and psychiatric illness in a population of pregnant substance-dependent patients?	Study finds high rate of smoking (88%) in their sample and smoking (yes/no) was correlated with current symptoms of anxiety or depression, but amount of smoking (# of cigarettes) did not correlate with presence psychiatric illness; the rate of use of nonopioid substance use did not differ between women who smoked and those who did not	Cross-sectional nature of the data precludes mechanisms from being assessed; self-report of smoking used with no biologic confirmations; assessment of psychiatric illness only performed at treatment entry; sample size is small and unequal group size impact power to detect differences

Described below are the effects of dual diagnosis in pregnant women on (a) illness severity and functional impairment, and (b) treatment adherence/completion.

#### Illness Severity and Functional Impairment

In order to assess illness severity and functional impairment in pregnant women diagnosed with OUD and comorbid psychiatric disorders, a few of the studies utilized the Addiction Severity Index (ASI), a semi-structured interview that evaluates pretreatment impairment on seven domains (medical, employment, drug, alcohol, legal, family/social, and psychiatric) and assigns a composite score to each domain [28]. In contrast, Haller et al. used self-report questionnaires and non-SCID structured interviews to assess severity of illness, and Chisolm et al. did not report a measure for illness severity [14, 22••]. Every study using ASI reported higher psychological impairment scores in pregnant women with both OUD and psychiatric comorbidity than in pregnant women with OUD only. These studies also found that women with both OUD and psychiatric comorbidity had higher composites scores of family/social impairments.

Eggleston et al. and Moylan et al. investigated the relationship between PTSD and SUD in pregnancy by using the SCID [15, 16••]. Moylan et al. found that women with both SUD and PTSD exhibited greater impairment in the ASI Psychological and Family/Social Domains as compared to women with SUD only or women with both SUD and non-PTSD Axis I disorders. Eggleston et al. found that pregnant women with both SUD and PTSD had higher rates of the following conditions within the ASI Psychological Impairment Domain (as compared to pregnant women with SUD only or SUD and a non-PTSD Axis I disorder): suicidal ideation (lifetime adjusted and within the last 30 days), suicide attempts (lifetime adjusted), and trouble controlling violence (lifetime adjusted and in the last 30 days). Pregnant women with both SUD and PTSD also had higher rates of the following conditions in the ASI Family/Social Domains: problematic relationships, homelessness, and illegal behaviors.

Pregnant women with SUD and copsychiatric disorders do not necessarily have more severe SUD at treatment entry. Four studies found no differences in the drug and alcohol use domains between pregnant women with and without psychiatric comorbidities, suggesting that substance use disorder severity is comparable between participants with and without psychiatric comorbidity [15, 16••, 21, 30]. It should be noted that part of what is being seen in the lack of severity in the SUD domain relates to a relative ceiling effect seen in patients with OUD where they are all so severe that it is difficult to find differences in this domain. In contrast, Benningfield et al. found a difference in the drug use domain, which might be explained by the study’s use of the MINI to screen for psychiatric symptoms. Specifically, they reported more severe substance use in patients with major depressive disorder, generalized anxiety disorder, hypomania, dysthymia, PTSD, and suicidality [18]. Neither Eggleston et al. nor Moylan et al. found higher drug and alcohol composite scores or ASI individual items among pregnant women with PTSD as compared to women without PTSD [15, 16••]. Haller et al. found that patients with greater psychopathology had similar substance use patterns when compared to patients with lower psychopathology, with severity evaluated using MMPI, SIPD-R, and MCMI-II self-report questionnaires [14]. The researchers used psychopathology based clusters to create three groups ranked by severity of psychopathology from low (i.e., SUD only, “benign” psychiatric comorbidities identified outside the SUD) to moderate (Axis II and SUD) to high (Axis I disorders and SUD), based on MCMI-II Base Rate Scores. Benningfield et al. found no significant differences in substance use between participants in four groups (with putative diagnoses based on the MINI diagnostic tool): depressive disorders and SUD; anxiety disorders and SUD; anxiety, depressive disorders, and SUD; and SUD only. Based on the studies by Fitzimmons et al., Eggleston et al., Moylan et al., and Haller et al., it appears that measuring SUD severity alone is insufficient to determine illness severity and functional impairment in pregnant women entering OUD treatment.

The studies reviewed had differing rates of poly-substance use in pregnant women with OUD with or without comorbid psychiatric disorders, and not all the studies reviewed addressed this parameter. Eggleston et al. found no difference in poly-substance use at treatment entry, other than for tobacco use, which was higher in women with comorbid PTSD, but not other non-SUD psychiatric illnesses [16••]. Chisolm et al. found that pregnant women with OUD and psychiatric comorbidity were more likely to smoke tobacco than those without psychiatric comorbidity, but in this study, PTSD was not analyzed separately from other non-SUD-psychiatric illnesses [22••]. Benningfield et al. found statistically significant difference in ASI Drug use domain for patients with major depressive disorder, generalized anxiety disorder, hypomania, dysthymia, and PTSD, when compared to patients with OUD only, but did not discuss rates of nicotine use [23]. Moylan et al. reported 89% poly-substance use in their sample, and 76% nicotine use but did not discuss whether rates were different in subjects with and without non-SUD psychiatric illnesses [15].

#### Treatment Adherence and Completion

A literature review by Greenfield et al. found that pregnant women with SUD and psychiatric comorbidities have lower treatment retention rates than women without psychiatric comorbidities [29]. This literature review had similar results when focusing on the subpopulation of pregnant women with OUD and psychiatric comorbidities.

Haller et al. reported that participants with the most severe psychopathology (those with SUD and Axis I disorders, including patients with psychotic illness) had the highest dropout rates and fastest attrition rate, with only 33% completing treatment. The group with the least severe psychopathology (participants with SUD only) had a lower dropout rate (57% completed treatment). Interestingly, the group with moderate psychopathology (participants with SUD and Axis II disorders) had the lowest dropout rate (76% completed treatment) [14]. The group with the least severe psychopathology may have had relatively high dropout rates because they needed fewer services, while the group with the most severe psychopathology’s relatively high dropout rate could be due to a lack of appropriate services. Surprisingly, a large percentage of patients with personality disorders completed treatment despite generally being considered “difficult to treat.” However, the program was specifically designed for patients with personality disorders, including a “holding environment” [14].

The literature presents conflicting results on the correlation between cooccurring psychiatric disorders and treatment adherence. Fitzsimmons et al. studied a population undergoing methadone treatment and found a correlation between anxiety diagnoses and better treatment adherence and retention (demonstrated by greater number of days in treatment, longer time in programming on treatment days, and fewer positive urine tests) as compared to patients with primary depression diagnoses or no anxiety or depression diagnoses [26••]. In contrast, Benningfield et al. found that patients with putative depressive diagnoses were relatively more likely to be retained in treatment than patients without putative depressive diagnoses, and patients with putative anxiety diagnoses were more likely to leave treatment than patients without putative anxiety diagnoses [17].

Moylan et al., surprisingly, found no statistically significant difference in treatment retention between groups of patients with and without PTSD (17.3 vs. 15.7 days) [15]. Similarly, Eggleston et al. found no statistically significant differences between urines drug screens of subjects with SUD only, SUD and Axis I Disorders, and subjects with PTSD and SUD [16••]. However, more research into the relationship between cooccurring PTSD and treatment retention is needed, especially given the frequency with which PTSD occur alongside other psychiatric comorbidities as discussed above.

## Discussion

Per this literature review, 25–33% of pregnant women with OUD have a psychiatric comorbidity, with depression and anxiety being especially common. However, these results must be interpreted with caution. Only one study reviewed by the authors (Benningfield et al. 2010) met all the following criteria: reported the prevalence rates as primary research outcome, used a validated screening method, and occurred in the last 10 years. While the authors report a range of prevalence rates from other studies, they must be interpreted with caution given inconsistency in screening methods and research questions. Furthermore, some studies reported prevalence rates prior to treatment initiation, while others reported screening rates after treatment initiation. Future studies should report prevalence rates for both time periods. Additionally, the authors suspect that rates of cooccurring psychiatric conditions may be underreported during screening, especially considering stigma and fear of reporting requirements to authorities (such as Departments of Child Services). The environment within which screening is conducted, including empathy exhibited by the researcher or clinician, may affect accuracy of reported results. Due to all the limitations discussed above, the rates reported in this review must be viewed with caution. There is a clear need for a more comprehensive population-based study on pregnant women with OUD to determine rates of various comorbid psychiatric conditions in this population.

Based on this literature review, it appears that that complications brought upon by psychiatric comorbidities are not reflected simply in greater severity of opioid use disorder but are also reflected in the degree to which the woman’s psychiatric, family, and social functioning is impaired. The data reviewed further suggests cooccurring psychiatric morbidities impact treatment adherence in pregnant women with OUD and that retaining pregnant women with OUD in treatment improves with integrated treatment of their addictive and nonaddictive mental illnesses. Interestingly, several studies found that women with OUD and diagnoses of depressive disorders, anxiety disorder, and PTSD were more likely to adhere to treatment than women with OUD only [12, 13, 18, 27]. These higher rates of adherence for women with anxiety and depression, and even PTSD, were found at treatment settings that provided integrated obstetrics and addiction treatment along with treatment for their cooccurring psychiatric illnesses and increased social support, which may suggest that the treatment settings that address these cooccurring illnesses may differentially benefit women diagnosed with these disorders. Other possible hypotheses for this finding include greater perceived need for care, greater familiarity with the healthcare system, and less stigma regarding being diagnosed with a psychiatric illness. However, it is difficult to have confidence in these results due to several design flaws in the studies including small sample sizes ranging from 78 to 174, use of different instruments to diagnose the comorbid diagnosis, variable time courses, and assessed adherence by different measures. Also, two of the studies did not have treatment adherence as the primary goal of the study, which is of significant concern [21].

Previous work has demonstrated that treatment for pregnant women with OUD is most effective when it is gender-specific, provides comprehensive care, and addresses the unique barriers these women face, and this review confirms this and adds that the dimension of comorbid mental illness is a particularly important area to attend to [29–31]. The authors recommend that paying special attention to the specific needs of the patients’ psychiatric disorder is vital to retention and both treatment type (individual or group) and intensity (inpatient, partial program, intensive outpatient program, or weekly group therapy) will be differentially effective for patients with different mental illnesses. Patients with personality disorders, for example, can be engaged in and complete treatment if the program considers their pathology and a developmentally appropriate treatment approach [14]. The many social and family impairments that complicate the lives of pregnant women with PTSD, and the greater difficulty in trusting treatment providers, also necessitate special attention to trauma-informed care and some modulation of the usual confrontational style of many SUD programs. In particular, pregnant women with SUD’s tend to experience a significant amounts of guilt, shame, and fear of DCS removal of their children, which is both a substantial barrier to entering treatment, but also to remaining in treatment, and thus a nonjudgmental, nonpunitive environment is particularly important for treating this population [5]. These considerations cannot be attended to if providers that treat pregnant women are not aware of patients’ psychiatric and/or addictive illnesses; thus, this review adds poignancy to the need to integrate screening and diagnosis of these disorders to routine prenatal care of all women. Additionally, the rates of poly-substance use were found to be extremely high (up 93% if nicotine is included, and up to 86% when not), and thus treatment of women with OUD must also include specific treatments for the other SUD, including nicotine, the cravings for which will not be treated by the MAT that often helps manage OUD [16••, 26••].

The authors see a pressing need for more studies with larger sample sizes, validated diagnostic tools, and longitudinal study methods. Studies reporting treatment adherence should include measures such as days in treatment, time spent in treatment each day, and urine drug screen results during treatment. Also, measures of progression through stages of change, measures of social, family, and relationship functioning, and changes in engagement with general medical care would provide more details about how subjects are affected by the treatment and how to optimize programing. Research to evaluate different types of therapy, effects of environmental factors, and medication management approaches in this population are also needed.

This review and other studies recommend integrating prenatal care and addictions treatment and psychiatric care in one setting is preferred for pregnant women [32]. Further research is still needed to compare providing prenatal care in a dual diagnosis setting or address psychiatric illnesses in an Ob-Gyn setting. Benefits to providing the care in a dual diagnosis setting might include the greater availability of dual diagnosis treatment and continuity of care following delivery when there is a reduction in contact with an Ob-Gyn provider. Benefits to providing the treatment in an Ob-Gyn setting include lower stigma and patient comfort for some patients. Additionally, the authors suggest including a measure of participants’ experience with and ownership of their treatment. Some ethnographic studies that give a greater and more detailed picture of the women’s experience with treatment providers and programs would be essential for designing patient-centered and empowering programming. Such studies exist for the general population of pregnant women with SUD, but few are specific to pregnant women with OUD and psychiatric comorbidities [33–36].

In addition, although a large randomized controlled trial have established the equivalence of methadone and buprenorphine for OUD treatment during pregnancy, the authors did not identify any randomized controlled trials that evaluated different types of psychiatric treatments (medication or therapy) for cooccurring psychiatric disorders pregnant women experience [37]. There is limited guidance on combining different psychiatric medications for treatment of co-occurring psychiatric illnesses in the setting of either methadone or buprenorphine in pregnant women. Given that a large percentage of pregnant women with OUD may already be taking psychiatric medication, and because psychiatric medications can impact metabolism, response to treatment, birth, and neonatal outcomes, more research into medication management for this subpopulation is needed.

## Conclusions

On balance, this review highlights the complexity facing many providers who work with pregnant women with OUD as these women tend to have high rates of psychiatric comorbidity and multiple SUD’s along with complicated social and family dimensions. More studies are needed of the comorbid psychiatric conditions affecting pregnant women with OUD to create an evidence base from which to draw when establishing treatment programs to address the needs of this population. Assessment of psychiatric comorbidity and SUD should be a part programing with concurrent treatment; however, precise recommendations on treatment setting, therapy modalities, and medication management all require further research.

## Appendix.

Database: Ovid MEDLINE(R) <1946 to September Week 4 2016>, Ovid MEDLINE(R) Daily Update <October 03, 2016>, Ovid

MEDLINE(R) In-Process & Other Non-Indexed Citations <October 03, 2016>

Search Strategy:

1. exp Demography/ (1147703)
2. exp population characteristics/ (1650409)
3. exp Pregnancy Outcome/ or exp Pregnancy Rate/ or exp Pregnancy/ or exp Pregnancy, Unplanned/ or exp Pregnancy,
4. Unwanted/ or exp Pregnancy Complications/ or exp Pregnancy, Prolonged/ or exp Time-to-Pregnancy/ (838186)
5. exp Opiate Substitution Treatment/ or exp Opioid-Related Disorders/ (21716)
6. exp Geographic Mapping/ (664)
7. exp Geography, Medical/ (1597)
8. 1 or 2 or 5 or 6 (1651533)
9. 3 and 4 and 7 (161)
10. 3 and 4 (1075)
11. 9 not 8 (914)
12. limit 10 to comment (33)
13. 10 not 11 (881)
14. limit 12 to (English language and humans) (719)
